# Spatial-Multiplexed Four-Channel Optical Amplification via Multiple Four-Wave Mixing in a Double-Λ Atomic System

**DOI:** 10.3390/nano16030184

**Published:** 2026-01-29

**Authors:** Xin Li, Dan Song, Yu-Xia Fan, Rong Miao, Dan Wang, Bao-Dong Yang, Hai-Tao Zhou, Jun-Xiang Zhang

**Affiliations:** 1 School of Physics and Electronic Engineering, Shanxi University, Taiyuan 030006, China; lixin27@sxu.edu.cn (X.L.); songdan@sxu.edu.cn (D.S.); fanyuxia@sxu.edu.cn (Y.-X.F.); miaorong@sxu.edu.cn (R.M.); wangdan63@sxu.edu.cn (D.W.); ybd@sxu.edu.cn (B.-D.Y.); 2 Collaborative Innovation Center of Extreme Optics, Shanxi University, Taiyuan 030006, China; 3 School of Physics, Zhejiang University, Hangzhou 310027, China

**Keywords:** multiple four-wave mixing, optical amplification, orbital angular momentum, vortex beam, hot atoms

## Abstract

Optical amplification and spatial multiplexing technologies have important applications in quantum communication, quantum networks, and optical information processing. In this paper, based on the non-reciprocal amplification of a pair of co-propagating conjugate four-wave mixing (FWM) signals induced by a one-way pump field in a double-Λ-type hot atomic system, we demonstrate spatially multiplexed multiple FWM processes by introducing a counter-propagating collinear pump field. This configuration enables simultaneous amplification of bidirectional four-channel FWM signals. Furthermore, when the injected signal and pump beams are modulated to Laguerre–Gaussian beams carrying different optical orbital angular momentum (OAM), the OAM of the pump beam is transferred to each amplified field. Through the tilted lens method, we experimentally demonstrate that the OAM of the amplified signal light remains identical to that of the original injected signal light. In contrast, the OAM of the other three newly generated FWM fields is governed by the angular momentum conservation law of their respective FWM processes, which enables the precise manipulation of the OAM for the other generated amplified fields. Theoretical analysis of the dynamical transport equation for the density operator in light–matter interaction processes fully corroborates the experimental results. These findings establish a robust framework for developing OAM-compatible optical non-reciprocal devices based on complex structured light.

## 1. Introduction

Optical amplification and spatial multiplexing technologies play a crucial role in overcoming the limits of information transmission capacity and enhancing information processing speed [1,2,3]. These technologies have wide-ranging applications in quantum communication and photonic quantum networks. A major challenge lies in achieving multi-channel and high-efficiency optical signal amplification while preserving the quantum properties of high-dimensional information encoding and signal transmission [4]. Orbital angular momentum (OAM), which corresponds to orthogonal spatial modes with different topological charges, theoretically offers infinite-dimensional information encoding resources. OAM-based spatial multiplexing technology can construct high-dimensional Hilbert spaces, providing a critical physical foundation for high-capacity quantum communication, parallel quantum computing, and complex quantum state simulation and manipulation [5,6,7,8]. However, traditional optical amplification technologies face challenges such as gain imbalance and mode crosstalk when simultaneously amplifying multiple channels and preserving OAM modes [9,10]. These limitations constrain their applicability to complex quantum systems requiring high-dimensional and high-fidelity operation.

Four-Wave Mixing (FWM) is a parametric process originating from the third-order nonlinear polarization of media, exhibiting unique characteristics including low noise, phase sensitivity, tunable bandwidth, and the capacity to generate quantum-correlated photon pairs [11,12,13,14,15]. As such, it has become a core nonlinear technology for achieving near-noiseless optical amplification [16,17], all-optical wavelength conversion [18,19], all-optical quantum gate operations [20,21], and deterministic quantum light sources [22,23]. However, traditional FWM systems driven by a single pump field face fundamental limitations in channel number scalability and OAM mode integration, which fall short of meeting the evolving demands of photonic quantum information technology for higher-dimensional and more complex architectures [24,25]. Recent years have witnessed significant progress in FWM based on alkali metal atoms (such as rubidium and cesium). The incorporation of electromagnetically induced transparency (EIT) enables the formation of highly coherent dark states near atomic resonance transition frequencies while simultaneously enhancing nonlinear optical responses. This breakthrough has opened new avenues for developing high-efficiency and low-noise FWM processes [26,27,28,29]. The applications of FWM have expanded from initial single-channel optical amplification and correlated photon pair generation [30,31,32] to the preparation of multiple correlated light beams through cascaded or parallel FWM configurations [33,34]. Nevertheless, most research efforts have predominantly focused on intensity or frequency multiplexing approaches [35,36], with comparatively limited attention devoted to the cooperative amplification and precise spatial mode manipulation of the FWM fields.

As a prototypical multi-level system, the double-Λ atomic energy level configuration demonstrates pronounced nonlinear optical phenomena and exceptional controllability under the coherent interaction of multiple light fields, thereby serving as an ideal platform for realizing efficient multi-channel FWM processes [37,38,39]. By introducing coupling fields and control fields at distinct transition energy levels, the atomic system enables the construction of dual or multiple EIT windows. This not only effectively suppresses the linear absorption of probe light across multi-frequency channels but also concurrently enhances the third-order nonlinear susceptibility in the vicinity of multiple resonant frequency points, thereby facilitating the realization of multi-channel optical amplification based on FWM [40,41]. Moreover, the system opens a new avenue for the parallel processing and coherent manipulation of multi-dimensional photon quantum states, including those carrying OAM [42,43,44].

In this study, we propose and implement a four-channel optical amplification transmission scheme utilizing multiple FWM in a double-Λ cesium atomic system. Under the action of a single pump field, the nonreciprocal amplification (NRA) of co-propagating dual FWM fields is demonstrated by accounting for the Doppler effect of thermal atoms [45,46,47,48]. Subsequently, by introducing a counter-propagating collinear pump field, a spatially multiplexed multi-FWM process is established, enabling bidirectional four-channel FWM signal amplification. Furthermore, we use spiral phase plates (SPP) to modulate the signal and pump beams to high-order Laguerre–Gaussian (LG) beams carrying different OAM quantum numbers, then experimentally and theoretically investigate the transfer characteristics of OAM among various FWM fields driven by the double counter-propagating pump fields. This scheme not only surpasses the channel number limitations of conventional FWM techniques but also significantly enhances spatial multiplexing capacity through the integration of OAM degrees of freedom. It thereby provides a novel approach for constructing high-capacity, multi-dimensional optical quantum information processing systems.

## 2. Experimental Setup and Results Analysis

**Figure 1:**
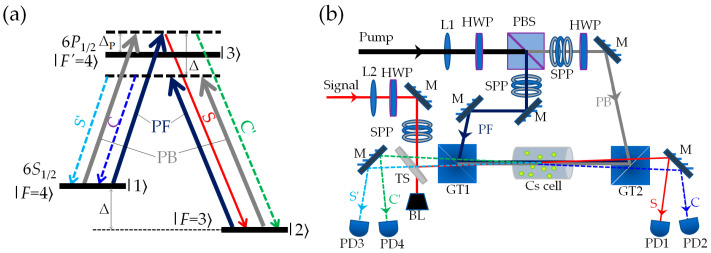
(**a**) Energy levels of the D1 line of the ^133^Cs atom involved in multiple FWM in a double-Λ scheme. Δ_P_ is the frequency detuning of the pump light, and Δ ≈ 2π × 9.192 GHz is the ground-state energy splitting. (**b**) Schematic for the experimental setup. HWP, half wave plate; PBS, polarizing beam splitter; GT1 and GT2, Glan–Taylor splitter with extinction ratio 10^5^:1; L1 and L2, planoconvex lens with focal length *f*_1_ = 750 mm and *f*_2_ = 500 mm; TS, beam splitter with reflectivity of 1%; M, 45° mirror; SPP, spiral phase plate; BL, beam block; PD1–PD4, photo detectors.

The energy level diagrams of the atoms are shown in Figure 1a. An Λ-type atomic system includes two ground states |1⟩ and |2⟩ and one excited state |3⟩. This system can be realized in the D1 line of the ^133^Cs atoms with |6*S*_1/2_, *F* = 4⟩, |6*S*_1/2_, *F* = 3⟩, and |6*P*_1/2_, *F*′ = 4⟩ acting as states |1⟩, |2⟩ and |3⟩, respectively. The frequency difference between the two lower levels is Δ ≈ 2π × 9.192 GHz. The two transitions |1⟩→|3⟩ and |2⟩→|3⟩ are simultaneously driven by two strong counter-propagating pump fields (forward pump field PF and backward pump field PB with the same frequency *ω*_P_) with detunings Δ_P_ = *ω*_P_ − *ω*_31_ and Δ_P_ − Δ. The transition |3⟩ to |2⟩ is also coupled by a weak signal light S with frequency *ω*_S_ and detuning Δ_S_ = *ω*_S_ − *ω*_32_. The multiple FWM based on the double-Λ scheme [30] can be satisfied when Δ_S_ = Δ_P_, that is, while S is being amplified, three new FWM fields are simultaneously generated, which are denoted as C, S′, and C′.

The schematic diagram of our experimental setup is shown in Figure 1b. We use two grating-feedback external-cavity diode lasers (Toptica DL100, with the center wavelength of 894.5 nm, the linewidth of 1 MHz, and the frequency tuning range of 30 GHz), each shaped to a fundamental mode Gaussian beam (TEM_00_) by optical fibers, as the strong pump beam and the weak signal beam, respectively. The strong pump beam is divided into two parts, PF and PB, which are reflected to the *L* = 25 mm-long Cs cell with vertical polarization through two Glan–Taylor prisms (GT1 and GT2, with the extinction ratio of 10^5^:1) in a collinear opposite direction. The weak signal beam S is reflected by a beam splitter (TS, with reflectivity 1%), and is then passed through the Cs cell with horizontal polarization to overlap with the PF at a small angle *θ* (*θ* ≈ 0.23°). The *e*^−2^ beam widths of PF, PB, and S at the center of the cell are approximately 0.69 mm, 0.68 mm, and 0.49 mm, respectively. The cell temperature is stabilized at *T*_cell_ = 100 °C. The forward FWM fields S and C are detected by PD1 and PD2, and the backward FWM fields S′ and C′ are detected by PD3 and PD4, respectively.

**Figure 2:**
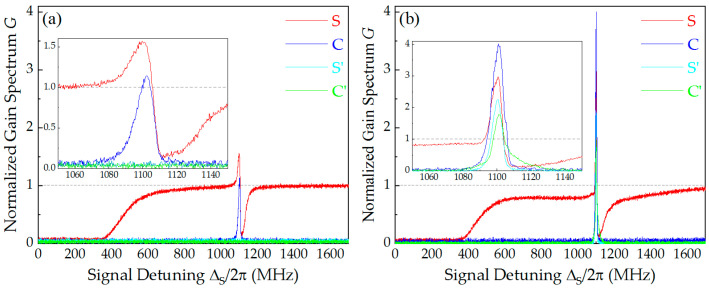
(Color online) The normalized gain spectrum *G* detected by PD1–PD4 versus signal detuning Δ_S_ with different pump excitation: (**a**) only the forward pump PF (*P*_PF_ = 250 mW, *P*_PB_ = 0); (**b**) both PF and PB are present (*P*_PF_ = 250 mW, *P*_PB_ = 200 mW). The experimental parameters are: *P*_S0_ = 200 μW, *T*_cell_ = 100 °C, and Δ_P_ = 2π × 1.1 GHz.

Figure 2 presents the normalized gain spectra under varied pumping configurations. The optical gain *G*_i_ of the FWM process is defined as the ratio of the output power *P*_i_ to the input signal power *P*_S0_ [30], i.e., *G*_i_ = *P*_i_/*P*_S0_ (i = S, C, S′, and C′). With the injection of solely the forward PF and S into the Cs cell, a forward FWM process is initiated. This process is enabled by the fulfillment of the phase-matching condition within the forward Doppler-free geometry, which concurrently satisfies the criteria for NRA [47,48]. At the two-photon resonance (e.g., Δ_S_ = Δ_P_ ≈ 2π × 1.1 GHz), S is amplified, and a newly forward conjugate beam C is created in the same direction of its symmetry with the PF field, as shown by the red and blue curves in Figure 2a. At this time, no backward beams can be detected by PD3 and PD4, as shown by the light blue and green lines in the inset of Figure 2a. To quantitatively describe the nonreciprocity, here we introduce the contrast ratio *η* = (*G*_S(C)_ − *G*_S′(C′)_)/(*G*_S(C)_ + *G*_S′(C′)_) [46]. Obviously, along the two propagation directions of forward FWM signals, *η* is greater than 95% (along the S direction, *η* ≈ 98%; along the C direction, *η* ≈ 96%). If the intrinsic dark current of the detector is neglected, the contrast ratio on both propagation directions approaches 1. Furthermore, when both PF and PB are present, multiple FWM processes occur simultaneously (see Figure 1). Along the opposite directions of the S and C, two new FWM beams denoted S′ and C′ are generated at the two-photon resonance, as shown by the light blue and green curves in Figure 2b. By comparing Figure 2a,b, it can be seen that on the basis of forward FWM (only PF injected), when the PB is introduced, the gain of the forward FWM fields *G*_S_ increases from 1.5 to 3, while *G*_C_ increases from 1.2 to 3.9. At this time, the gain of newly generated backward FWM fields *G*_S′_ ≈ 2.3 and *G*_C′_ ≈ 1.8. Actually, *G*_S_ should exceed *G*_C_, which should be satisfied with *G*_C_ = *G*_S_ − 1 [15,30]. Here, *G*_S_ is less than *G*_C_ primarily because the frequency of S is close to the Doppler absorption range of thermal atoms, resulting in greater absorption loss during its propagation in the Cs cell after amplification. It is noteworthy that when Δ_S_ is scanned near the atomic resonance center, the higher vapor temperature (*T*_cell_ = 100 °C) leads to increased atomic number density and enhanced thermal motion effects within the Cs cell. Consequently, the week S injected the Cs cell (*P*_S0_ = 200 μW) should be completely absorbed by atoms over a broad Doppler-broadened range (Δ_S_ is from about −400 MHz to 400 MHz). As for the strong absorption of S near ~1120 MHz detuning, it is mainly caused by the Raman absorption. Owing to the competition of EIT, FWM, and Raman absorption, the gain spectrum of S near two photon resonances appears to have a slight dispersive character [32], as illustrated by the red curves in Figure 2.

**Figure 3:**
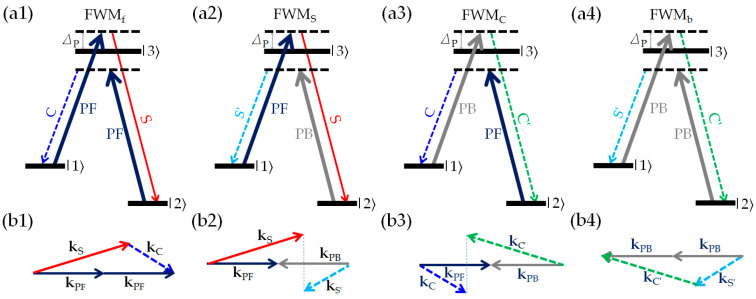
The energy-level transition schemes (**a1**–**a4**) and corresponding phase-matching diagrams (**b1**–**b4**) of the multiple FWM interactions induced by the two counter-propagating pump fields. **k** is the wave vector of the beam in the atomic medium.

Actually, the four-channel optical amplification of Figure 2b is the result of the mutual superposition and enhancement of multiple FWM processes, which include four interaction processes between light and atoms, as shown in Figure 3. The first FWM process (named FWM_f_) is induced under the action of forward PF and S fields [see Figure 3(a1)]: The atom, initially in the ground state |1⟩, first absorbs a forward pump photon PF and is stimulated to the excited state |3⟩, and then emits a forward signal photon S and jumps to another ground state |2⟩. Immediately after, the atom absorbs a forward pump photon PF and is stimulated to the excited state |3⟩, and finally emits a forward conjugate photon C, and finally jumps back to the ground state |1⟩. FWM_f_ can be described as $|1\rangle \overset{\mathrm{PF}}{\underset{A}{\to }}|3\rangle \overset{S}{\underset{E}{\to }}|2\rangle \overset{\mathrm{PF}}{\underset{A}{\to }}|3\rangle \overset{C}{\underset{E}{\to }}|1\rangle$ (*A* and *E* represent the absorption and emission of the atom, respectively), with energy conservation relation 2*ω*_P_ = *ω*_S_ + *ω*_C_ and phase matching relation 2**k**_PF_ = **k**_S_ + **k**_C_, see Figure 3(b1). When considering the backward PB, the forward amplified S and generated C can, respectively, serve as seed beam, interacting with the two counter-propagating pump fields (PF and PB) to generate two additional FWM processes, named as FWM_S_ and FWM_C_, as shown in Figure 3(a2,a3). The interaction process of FWM_S_ can be expressed as $|1\rangle \overset{\mathrm{PF}}{\underset{A}{\to }}|3\rangle \overset{S}{\underset{E}{\to }}|2\rangle \overset{\mathrm{PB}}{\underset{A}{\to }}|3\rangle \overset{S^{\prime }}{\underset{E}{\to }}|1\rangle$ with 2*ω*_P_ = *ω*_S_ + *ω*_S′_ and **k**_PF_ + **k**_PB_ = **k**_S_ + **k**_S′_ [see Figure 3(b2)], and that of FWM_C_ can be expressed as $|2\rangle \overset{\mathrm{PF}}{\underset{A}{\to }}|3\rangle \overset{C}{\underset{E}{\to }}|1\rangle \overset{\mathrm{PB}}{\underset{A}{\to }}|3\rangle \overset{C^{\prime }}{\underset{E}{\to }}|2\rangle$ with 2*ω*_P_ = *ω*_C_ + *ω*_C′_ and **k**_PF_ + **k**_PB_ = **k**_C_ + **k**_C′_ [see Figure 3(b3)]. Finally, the newly generated S′(C′) and PB contribute to the backward FWM (named as FWM_b_), expressed as $|2\rangle \overset{\mathrm{PB}}{\underset{A}{\to }}|3\rangle \overset{S^{\prime }}{\underset{E}{\to }}|1\rangle \overset{\mathrm{PB}}{\underset{A}{\to }}|3\rangle \overset{C^{\prime }}{\underset{E}{\to }}|2\rangle$ with 2*ω*_P_ = *ω*_S′_ +*ω*_C′_ and 2**k**_PB_ = **k**_S′_ +**k**_C′_, as shown in Figure 3(a4,b4).

If the fundamental Gaussian optical fields injected into the Cs vapor cell are replaced by vortex beams carrying *l*ℏ OAM (*l* is the OAM quantum number), the topological charge can also be transferred into the generated FWM beams through multiple phase-matched FWM interactions, which is in accordance with the principle of OAM conservation [9,10]. That is, the rules should be 2*l*_PF_ = *l*_S_ + *l*_C_ for FWM_f_, *l*_PF_ + *l*_PB_ = *l*_S_ + *l*_S′_ for FWM_S_, *l*_PF_ + *l*_PB_ = *l*_C_ + *l*_C′_ for FWM_C_, and 2*l*_PB_ = *l*_S′_ + *l*_C′_ for FWM_b_. Furthermore, to validate this inference, we employed a spiral phase plate (SPP, which includes one vortex phase plate and two quarter-wave plates) to modulate PF, PB, as well as S into Laguerre–Gaussian (LG) beams [see Figure 1b], respectively, and observed the output pattern of the four amplified FWM fields by a camera.

**Figure 4:**
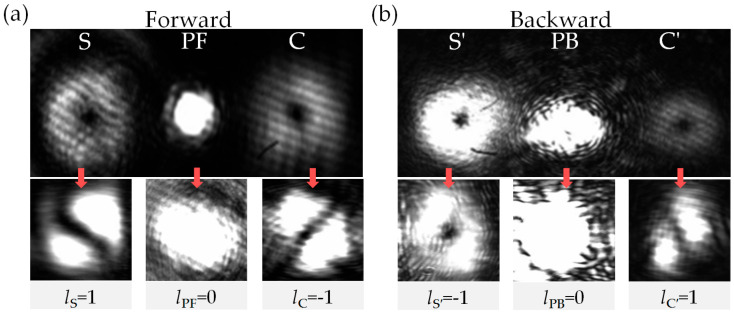
The patterns of the (**a**) forward output beams and the (**b**) backward output beams captured by a camera when the OAM quantum numbers of input beams are: *l*_S_ = 1 and *l*_PF_ = *l*_PB_ = 0. The frequency detuning of the signal light is locked to Δ_S_ = Δ_P_ = 2π × 1.1 GHz. The other parameters are the same as in Figure 2.

When the injected S is modulated to LG_01_ mode with *l*_S_ = 1, and the two counter-propagating pump beams PF and PB are both in the TEM_00_ mode (*l*_PF_ = *l*_PB_ = 0), the four output FWM fields in both forward and backward directions exhibit LG_01_ modes, while the modes of PF and PB remain unchanged, as shown the top in Figure 4a,b. Using the tilted lens method [49,50], the OAM quantum numbers of the forward amplified FWM fields are: *l*_S_ = 1 and *l*_C_ = −1, see the bottom in Figure 4a, and those of the backward generated FWM fields are *l*_S′_ = −1 and *l*_C′_ = 1, see the bottom in Figure 4b. Obviously, the experimental results demonstrate that all four FWM processes adhere to the angular momentum conservation principle.

**Figure 5:**
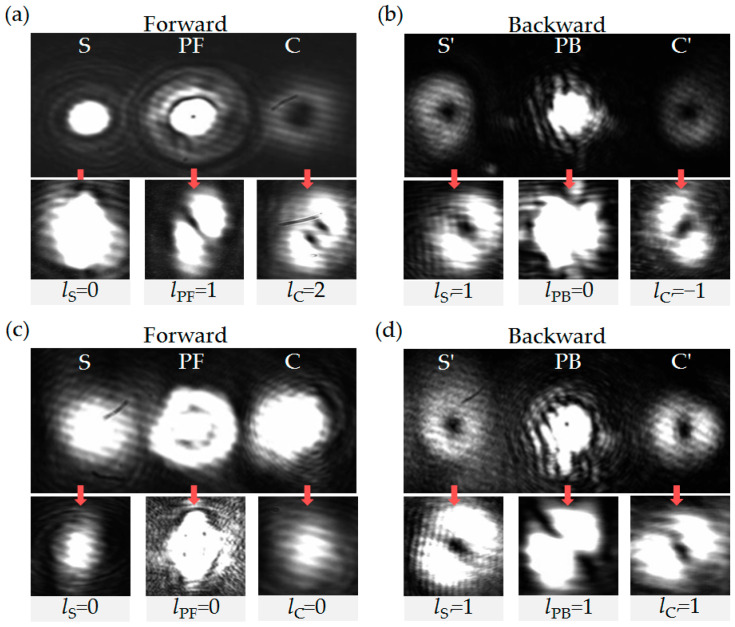
The patterns of the forward and backward output beams when modulating the OAM quantum numbers of the pump beams: (**a**,**b**) *l*_PF_ = 1, and *l*_S_ = *l*_PB_ = 0; (**c**,**d**) *l*_PB_ = 1, and *l*_S_ = *l*_PF_ = 0. The other parameters are the same as in Figure 4.

Actually, in each complete four-wave mixing process, while atoms absorb two pump photons and emit a pair of conjugate photons, OAM is simultaneously transferred from the pump photons to the amplified photons, in which the topological charge number transferred to each four-wave mixing light field depends on the original topological charge number of the injected signal light. Taking *l*_PF_ = 1, *l*_PB_ = 0 as an example, when the pattern of the injected S is TEM_00_, it is found that the OAM quantum number of the amplified S remains unchanged with *l*_S_ = 0, and that of the generated C is modulated to *l*_C_ = 2 according to angular momentum conservation in FWM_f_, as shown in Figure 5a. At the same time, the backward generated S′ (C′) is changed to LG_01_ with *l*_S′_ = 1 (*l*_C′_ = −1) by the process of FWM_S_ (FWM_C_), as shown in Figure 5b. As for the FWM_b_, the angular momentum conservation is also satisfied. Similarly, for the case of *l*_PF_ = 0, *l*_PB_ = 1, the forward S and C are the same OAM quantum number with *l*_S_ = *l*_C_ = 0, and those of the backward S′ and C′ are the same with *l*_S′_ = *l*_C′_ = 1, as shown in Figure 5c,d.

## 3. Theoretical Simulation of OAM Transfer

In order to quantitatively describe the dynamic behavior of OAM conversion in the multiple FWM processes, here we give the theoretical simulation using a semiclassical approach. See Figure 1a, under the condition of two counter-propagating pump fields, the Hamiltonian of the system can be shown as:

(1) $$\begin{array}{l} \overset{\wedge }{H}=\hbar \omega_{1}|1\rangle \langle 1|+\hbar \omega_{2}|2\rangle \langle 2|+\hbar \omega_{3}|3\rangle \langle 3|\\ -\hbar \left[\Omega_{\mathrm{PF}}e^{-i\omega_{P}t}+\Omega_{\mathrm{PB}}e^{-i\omega_{P}t}+\Omega_{C}e^{-i\omega_{C}t}+\Omega_{S^{\prime }}e^{-i\omega_{S_{\prime }}t}\right]|3\rangle \langle 1|\\ -\hbar \left[\Omega_{\mathrm{PF}}e^{-i\omega_{P}t}+\Omega_{\mathrm{PB}}e^{-i\omega_{P}t}+\Omega_{S}e^{-i\omega_{S}t}+\Omega_{C^{\prime }}e^{-i\omega_{C^{\prime }}t}\right]|3\rangle \langle 2|+h.c. \end{array},$$

where *ℏ* is the reduced Planck constant, Ω*_i_* = *μE_i_*/ *ℏ* (*i* = PF, PB, S, C, S′, C′) denotes the Rabi frequency of each field in multiple FWMs, and *h.c.* represents the Hermitian conjugate term. By inserting the Hamiltonian from Equation (1) into the Liouville–von Neumann equation (see Appendix A Equation (A1)), and using the rotating wave approximation (see Appendix A Equations (A2a)–(A2d)), we obtain the zero-order coupled equations (see Appendix A Equations (A3a)–(A3i)) and the first-order coupled equations (see Appendix A Equations (A4a)–(A4g)) for the density matrix elements. We then derive the expressions for the atomic coherence terms $\sigma_{31}^{\prime }$, $\sigma_{31}^{\prime \prime }$, $\sigma_{32}^{\prime }$, and $\sigma_{32}^{\prime \prime }$ under the first-order approximation of the four output optical fields. *A*_S(S′)_, *A*_C(C′)_, *B*_S(S′)_, and *B*_C(C′)_ are the parameters and their expressions see Appendix B.

(2a) $$\sigma_{31}^{\prime }=A_{S^{\prime }}\Omega_{S^{\prime }}+B_{S^{\prime }}{\Omega_{S}}^{*}+B_{S^{\prime }}{\Omega_{C^{\prime }}}^{*}$$

(2b) $$\sigma_{31}^{\prime \prime }=A_{C}\Omega_{C}+B_{C}{\Omega_{S}}^{*}+B_{C}{\Omega_{C^{\prime }}}^{*}$$

(2c) $$\sigma_{32}^{\prime }=A_{S}\Omega_{S}+B_{S}{\Omega_{S^{\prime }}}^{*}+B_{S}{\Omega_{C}}^{*}$$

(2d) $$\sigma_{32}^{\prime \prime }=A_{C^{\prime }}\Omega_{C^{\prime }}+B_{C^{\prime }}{\Omega_{S^{\prime }}}^{*}+B_{C^{\prime }}{\Omega_{C}}^{*}$$

Combining with the coupled Maxwell–Bloch equations describing the propagation of optical fields in the medium [51], the evolution equations for the four output optical fields along the propagation direction z of the medium are derived as:

(3a) $$\frac{\partial \Omega_{S}}{\partial z}=i\eta \sigma_{32}^{\prime }$$

(3b) $$\frac{\partial \Omega_{C}}{\partial z}=i\eta \sigma_{31}^{\prime \prime }$$

(3c) $$-\frac{\partial \Omega_{S^{\prime }}}{\partial z}=i\eta \sigma_{31}^{\prime }$$

(3d) $$-\frac{\partial \Omega_{C^{\prime }}}{\partial z}=i\eta \sigma_{32}^{\prime \prime }$$

where *η* = 3*N*_0_*λ*Γ/4π is the coupling constant (*N*_0_ is atomic number density, *λ* is wavelength, and Γ is attenuation coefficient). When numerically solving the above evolution equations, the initial conditions are set as: Ω_S_ (*z* = 0) = Ω_S0_ and Ω_C_ (*z* = 0) = 0, Ω_S′(C′)_ (*z* = *L*) = 0

As the fundamental non-vortex beam, the Gaussian beam has a light field distribution that satisfies the following expression [50]:

(4a) $$E\left(r\right)=A\sqrt{\frac{1}{\pi {w_{0}}^{2}}}\exp\left(-\frac{r^{2}}{{w_{0}}^{2}}\right)$$

The LG beam, which exhibits vortex characteristics and is capable of carrying orbital angular momentum, has the following light field expression:

(4b) $$E\left(r,\theta \right)=A\sqrt{\frac{2}{\pi {w_{0}}^{2}}}{\left(\frac{\sqrt{2}r}{w_{0}}\right)}^{{\left|l\right|}^{2}}\exp\left(-\frac{r^{2}}{{w_{0}}^{2}}\right){L_{p}}^{\left(2\left|l\right|+1\right)}\left(\frac{2r^{2}}{{w_{0}}^{2}}\right)\exp\left(il\theta \right)$$

where *A* is the amplitude of the light field, *w*_0_ is the beam waist, *r* is the radial coordinate, *θ* is the azimuthal coordinate, and *l* is the OAM quantum number.

To intuitively reveal the transfer mechanism of OAM, we numerically solved the aforementioned four partial differential equations [Equations (3a)–(3d)]. By substituting the OAM modes corresponding to S, PF, and PB under different conditions [Equations (4a) and (4b)] into the equation set, we finally obtained the intensity distributions and spiral phase structures of the four FWM fields under various scenarios.

**Figure 6:**
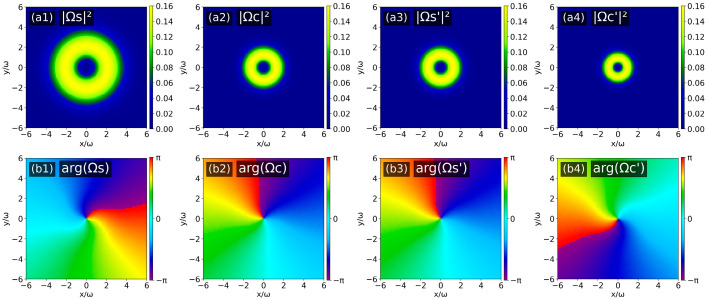
Intensity patterns (**a1**–**a4**) and phase patterns (**b1**–**b4**) of the four output optical fields (Ω_S(C)_ (*z* = 0), Ω_S′(C′)_ (*z* = *L*)) when *l*_S_ = 1 and *l*_PF_ = *l*_PB_ = 0.

To compare with the three different combinations of input OAM topological charges verified experimentally (the experimental configurations are shown in Figure 4 and Figure 5), we conducted corresponding theoretical simulations and obtained the intensity and phase patterns of the four output optical fields. First, for the first topological charge configuration: *l*_S_ = 1 and *l*_PF_ = *l*_PB_ = 0, Figure 6(a1)–(a4) shows that the intensity distributions of all four output fields exhibit typical annular structures with a phase singularity (dark core) at the center of the ring, indicating they are all vortex beams. The core criterion for the topological charge of OAM can be characterized by the azimuthal evolution of the helical phase distribution: a phase variation of 2π corresponds to a topological charge *l* = 1 when rotating counterclockwise around the optical axis for a full cycle, whereas a phase variation of −2π corresponds to *l* = −1 when rotating clockwise for a full cycle. As shown in Figure 6(b1)–(b4), the topological charges of the output S and C′ satisfy: *l*_S_ = *l*_C′_ = 1, and the topological charges of the output fields S′ and C satisfy: *l*_C_ = *l*_S′_ = −1.

**Figure 7:**
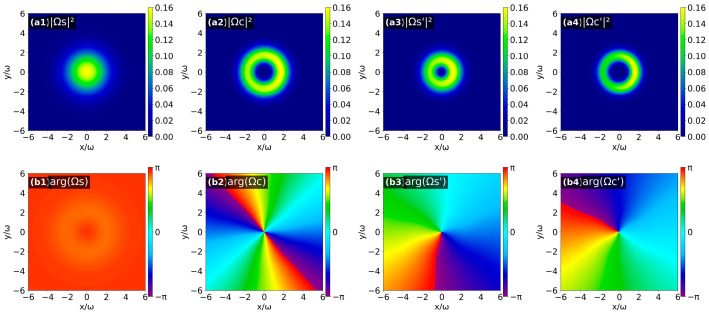
Intensity patterns (**a1**–**a4**) and phase patterns (**b1**–**b4**) of the four output optical fields (Ω_S(C)_ (*z* = 0), Ω_S′(C′)_ (*z* = *L*)) when *l*_PF_ = 1 and *l*_S_ = *l*_PB_ = 0.

**Figure 8:**
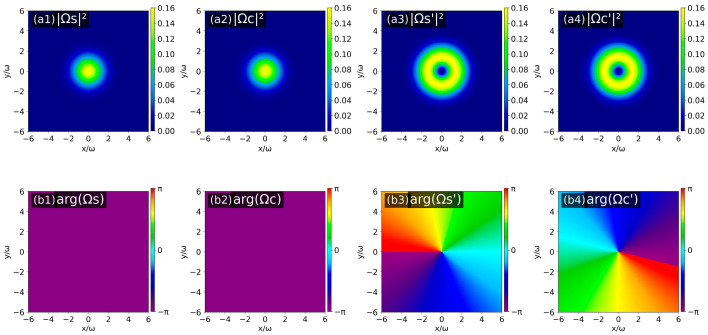
Intensity patterns (**a1**–**a4**) and phase patterns (**b1**–**b4**) of the four output optical fields (Ω_S(C)_ (*z* = 0), Ω_S′(C′)_ (*z* = *L*)) when *l*_PB_ = 1 and *l*_S_ = *l*_PF_ = 0.

Similarly, considering the second topological charge configuration (*l*_PF_ = 1, *l*_S_ = *l*_PB_ = 0) and the third topological charge configuration (*l*_PB_ = 1, *l*_S_ = *l*_PF_ = 0), the results are shown in Figure 7 and Figure 8, respectively. By combining the helical characteristics of the phase distributions and the phase evolution rule around the optical axis, the OAM topological charges of the four output fields under the two configurations are obtained as follows: for the second case: *l*_S_ = 0, *l*_C_ = 2, *l*_S′_ = 1 and *l*_C′_ = −1; for the third case: *l*_S_ = *l*_C_ = 0, *l*_S′_ = *l*_C′_ = 1.

The above three theoretical simulations are in excellent agreement with the experimental results and demonstrate that in our multiple FWM processes, whether it is FWM_f_ or FWM_S_, when an atom absorbs a pump photon and emits a signal photon S, it always transfers the same topological charge as the initial injected signal photon. Then, in accordance with the law of angular momentum conservation, the topological charge of the newly generated conjugate photon C or S′ is determined. Furthermore, the topological charge of C′ can also be determined by FWM_C_ or FWM_b_. Based on our experimental scheme, while achieving four-fold spatial multiplexing optical amplification, the OAM of each newly generated FWM signal can be effectively controlled by modulating the topological charge of the two counter-propagating pump fields.

## 4. Conclusions

In this study, a four-channel optical amplification scheme is experimentally demonstrated using multiple stimulated Raman FWM processes in hot Cs atoms. Under a single pump field, the generation of dual-channel conjugate non-degenerate Raman amplification signals requires FWM in the same direction and suppression in the opposite direction. However, by introducing a counter-propagating pump field, a pair of amplified signals can be generated in the opposite direction. Qualitative analysis reveals that the four-channel amplification arises from the mutual superposition and enhancement of multiple FWM processes. When the injected signal and pump lights are modulated into higher-order LG beams carrying OAM, both experimental and theoretical results confirm that the topological charge of the amplified signal photon remains unchanged, while the topological charges of the other three newly generated photons are determined by the conservation of angular momentum governed by the corresponding FWM processes. These findings enable precise manipulation of the OAM for multi-channel amplified fields by tuning the topological charge of the pump photons. The results establish a robust framework for developing OAM-compatible optical quantum devices based on complex structured light and hold significant potential for applications in high-capacity optical communication and high-dimensional signal processing.

## Data Availability

Data underlying the results presented in this paper are not publicly available at this time but may be obtained from the authors upon reasonable request.

## Appendix A. Bloch Equation

The evolution of the system is given by the Liouville–von Neumann equation [31]:

(A1) $$\overset{•}{\overset{\wedge }{\rho }}=-\frac{i}{\hbar }\left[\overset{\wedge }{H},\overset{\wedge }{\rho }\right]+L_{\gamma }\left[\overset{\wedge }{\rho }\right]$$

and the density matrix elements in the rotating frame are:

(A2a) $$\rho_{ii}=\sigma_{ii},$$

(A2b) $$\rho_{21}=\sigma_{21},$$

(A2c) $$\rho_{31}=\sigma_{31}e^{-i\omega_{0}t}+\sigma_{31}^{\prime }e^{-i\omega_{S^{\prime }}t}+\sigma_{31}^{\prime \prime }e^{-i\omega_{C}t},$$

(A2d) $$\rho_{32}=\sigma_{32}e^{-i\omega_{0}t}+\sigma_{32}^{\prime }e^{-i\omega_{S}t}+\sigma_{32}^{\prime \prime }e^{-i\omega_{C^{\prime }}t},$$

After applying the Rotating Wave Approximation (RWA), the zero-order coupled equations for the density matrix elements are as follows:

(A3a) $$\overset{•}{\sigma_{11}}+\overset{•}{\sigma_{22}}+\overset{•}{\sigma_{33}}=1,$$

(A3b) $$\overset{•}{\sigma_{11}}=\Gamma \sigma_{33}+i\Omega^{*}\sigma_{31}-i\Omega \sigma_{13},$$

(A3c) $$\overset{•}{\sigma_{22}}=\Gamma \sigma_{33}+i\Omega^{*}\sigma_{32}-i\Omega \sigma_{23},$$

(A3d) $$\overset{•}{\sigma_{21}}=-\Gamma_{21}\sigma_{21}+i\Omega^{*}\sigma_{31}-i\Omega \sigma_{23},$$

(A3e) $$\overset{•}{\sigma_{12}}=-\Gamma_{12}\sigma_{12}-i\Omega \sigma_{13}+i\Omega^{*}\sigma_{32},$$

(A3f) $$\overset{•}{\sigma_{31}}=-\Gamma_{31}\sigma_{31}+i\Omega \sigma_{21}+i\Omega N_{13},$$

(A3g) $$\overset{•}{\sigma_{13}}=-\Gamma_{13}\sigma_{13}-i\Omega^{*}\sigma_{12}-i\Omega^{*}N_{13},$$

(A3h) $$\overset{•}{\sigma_{32}}=-\Gamma_{32}\sigma_{32}+i\Omega \sigma_{12}+i\Omega N_{23},$$

(A3i) $$\overset{•}{\sigma_{23}}=-\Gamma_{23}\sigma_{23}-i\Omega^{*}\sigma_{21}-i\Omega^{*}N_{23},$$

Here, the complex decay rates are defined as Γ=γ, $\Gamma_{21}=\Gamma_{12}^{*}=i\delta$, $\Gamma_{31}=\Gamma_{13}^{*}=\gamma -i\Delta_{P}$, and $\Gamma_{32}=\Gamma_{23}^{*}=\gamma -i\delta -i\Delta_{P}$. We define that $\Omega =\Omega_{\mathrm{PF}}+\Omega_{\mathrm{PB}}$, $N_{13}=\sigma_{11}-\sigma_{33}$, $N_{23}=\sigma_{22}-\sigma_{33}$.

In a similar way, the first-order coupled equations for the density matrix elements are as follows:

(A4a) $$\begin{array}{ll} \overset{•}{\sigma_{21}}= & -\Gamma_{21}\sigma_{21}+\Omega^{*}\left(\sigma_{31}^{\prime }+\sigma_{31}^{\prime \prime }\right)e^{-i\delta t}+\left({\Omega_{C^{\prime }}}^{*}+{\Omega_{S}}^{*}\right)\sigma_{31}e^{-i\delta t}\\ & -\Omega \left(\sigma_{23}^{\prime }+\sigma_{23}^{\prime \prime }\right)e^{-i\delta t}-\left(\Omega_{C}+\Omega_{S^{\prime }}\right)\sigma_{23}e^{-i\delta t} \end{array},$$

(A4b) $$\overset{•}{\sigma_{31}}=-\Gamma_{31}\sigma_{31}+i\Omega N_{13}+i\left(\Omega_{C^{\prime }}+\Omega_{S}\right)\sigma_{21}e^{i\delta t},$$

(A4c) $$\overset{•}{\sigma_{31}^{\prime }}=-\Gamma_{31}\sigma_{31}^{\prime }+i\Omega_{\mathrm{PB}}\sigma_{21}e^{i\delta t}+i\Omega_{S^{\prime }}N_{13},$$

(A4d) $$\overset{•}{\sigma_{31}^{\prime \prime }}=-\Gamma_{31}\sigma_{31}^{\prime \prime }+i\Omega_{\mathrm{PF}}\sigma_{21}e^{i\delta t}+i\Omega_{C}N_{13}\to ,$$

(A4e) $$\overset{•}{\sigma_{32}}=-\Gamma_{32}\sigma_{32}+i\Omega N_{23}+i\left(\Omega_{C}+\Omega_{S^{\prime }}\right)\sigma_{12}e^{-i\delta t},$$

(A4f) $$\overset{•}{\sigma_{32}^{\prime }}=-\Gamma_{32}\sigma_{32}^{\prime }+i\Omega_{\mathrm{PF}}\sigma_{12}e^{-i\delta t}+i\Omega_{S}N_{23},$$

(A4g) $$\overset{•}{\sigma_{32}^{\prime \prime }}=-\Gamma_{32}\sigma_{32}^{\prime \prime }+i\Omega_{\mathrm{PB}}\sigma_{12}e^{-i\delta t}+i\Omega_{C^{\prime }}N_{23},$$

and

(A5a) $$N_{13}=\sigma_{11}-\sigma_{33}=\frac{\Gamma }{G}\left\{\begin{array}{l} \Gamma_{12}\Gamma_{13}\Gamma_{21}\Gamma_{31}\left(\Gamma_{23}+\Gamma_{32}\right)+2\left(\Gamma_{13}+\Gamma_{23}+\Gamma_{31}+\Gamma_{32}\right){\left|\Omega \right|}^{4}+\\ \left[\Gamma_{21}\Gamma_{31}\left(\Gamma_{13}+2\Gamma_{23}+\Gamma_{32}\right)+\Gamma_{12}\Gamma_{13}\left(\Gamma_{23}+\Gamma_{31}+2\Gamma_{32}\right)\right]{\left|\Omega \right|}^{2} \end{array}\right\},$$

(A5b) $$N_{23}=\sigma_{22}-\sigma_{33}=\frac{\Gamma }{G}\left\{\begin{array}{l} \Gamma_{12}\Gamma_{21}\Gamma_{23}\Gamma_{31}\left(\Gamma_{13}+\Gamma_{31}\right)+2\left(\Gamma_{13}+\Gamma_{23}+\Gamma_{31}+\Gamma_{32}\right){\left|\Omega \right|}^{4}+\\ \left[\Gamma_{12}\left(\Gamma_{23}+\Gamma_{31}\right)\Gamma_{32}+\Gamma_{21}\Gamma_{23}\left(2\Gamma_{31}+\Gamma_{32}\right)+\Gamma_{13}\left(\Gamma_{21}\Gamma_{23}+2\Gamma_{12}\Gamma_{32}\right)\right]{\left|\Omega \right|}^{2} \end{array}\right\},$$

(A5c) $$\begin{array}{ll} G= & \Gamma \Gamma_{12}\Gamma_{21}\left[\Gamma_{13}\Gamma_{23}\Gamma_{31}+\Gamma_{23}\Gamma_{31}\Gamma_{32}+\Gamma_{13}\left(\Gamma_{23}+\Gamma_{31}\right)\Gamma_{32}\right]\\ & +\left[4\Gamma +3\left(\Gamma_{12}+\Gamma_{21}\right)\right]\left(\Gamma_{13}+\Gamma_{23}+\Gamma_{31}+\Gamma_{32}\right){\left|\Omega \right|}^{4}\\ & +\left\{\begin{array}{l} 3\Gamma_{12}\Gamma_{21}\left(\Gamma_{13}+\Gamma_{31}\right)\left(\Gamma_{23}+\Gamma_{32}\right)+\Gamma \Gamma_{21}\left[\begin{array}{l} 4\Gamma_{23}\Gamma_{31}+\Gamma_{13}\left(\Gamma_{23}+\Gamma_{31}\right)\\ +\Gamma_{23}\left(\Gamma_{31}+\Gamma_{32}\right) \end{array}\right]\\ +\Gamma \Gamma_{12}\left[\Gamma_{32}\left(\Gamma_{23}+\Gamma_{31}\right)+\Gamma_{13}\left(\Gamma_{23}+\Gamma_{31}+4\Gamma_{32}\right)\right] \end{array}\right\}{\left|\Omega \right|}^{2}. \end{array}$$

## Appendix B. Coefficients of Equations (2a)–(2d)

The coefficients *A_i_* and *B_i_* of the expressions in Equations (2a)–(2d) are given by

(A6a) $$A_{S}=i\frac{N_{23}\Gamma_{31}\Gamma_{12}\Gamma_{13}\Gamma_{23}+N_{23}\Gamma_{31}\Gamma_{13}{\left|\Omega \right|}^{2}+N_{23}\Gamma_{13}\Gamma_{23}\Omega_{\mathrm{PB}}\Omega^{*}-N_{13}\Gamma_{31}\Gamma_{23}\Omega_{\mathrm{PF}}\Omega^{*}}{\Gamma_{31}\left[\Gamma_{31}\Gamma_{12}\Gamma_{13}\Gamma_{23}+\left(\Gamma_{31}\Gamma_{13}+\Gamma_{13}\Gamma_{23}\right){\left|\Omega \right|}^{2}\right]},$$

(A6b) $$A_{S^{\prime }}=i\frac{N_{13}\Gamma_{13}\Gamma_{21}\Gamma_{32}\Gamma_{23}+N_{13}\Gamma_{32}\Gamma_{23}{\left|\Omega \right|}^{2}+N_{13}\Gamma_{13}\Gamma_{23}\Omega_{\mathrm{PF}}\Omega^{*}-N_{23}\Gamma_{13}\Gamma_{32}\Omega_{\mathrm{PB}}\Omega^{*}}{\Gamma_{32}\left[\Gamma_{13}\Gamma_{21}\Gamma_{23}\Gamma_{32}+\left(\Gamma_{13}\Gamma_{23}+\Gamma_{23}\Gamma_{32}\right){\left|\Omega \right|}^{2}\right]},$$

(A6c) $$A_{C}=i\frac{N_{13}\Gamma_{13}\Gamma_{21}\Gamma_{32}\Gamma_{23}+N_{13}\Gamma_{32}\Gamma_{23}{\left|\Omega \right|}^{2}+N_{13}\Gamma_{13}\Gamma_{23}\Omega_{\mathrm{PB}}\Omega^{*}-N_{23}\Gamma_{13}\Gamma_{32}\Omega_{\mathrm{PF}}\Omega^{*}}{\Gamma_{32}\left[\Gamma_{13}\Gamma_{21}\Gamma_{23}\Gamma_{32}+\left(\Gamma_{13}\Gamma_{23}+\Gamma_{23}\Gamma_{32}\right){\left|\Omega \right|}^{2}\right]},$$

(A6d) $$A_{C^{\prime }}=i\frac{N_{23}\Gamma_{31}\Gamma_{12}\Gamma_{13}\Gamma_{23}+N_{23}\Gamma_{31}\Gamma_{13}{\left|\Omega \right|}^{2}+N_{23}\Gamma_{13}\Gamma_{23}\Omega_{\mathrm{PF}}\Omega^{*}-N_{13}\Gamma_{31}\Gamma_{23}\Omega_{\mathrm{PB}}\Omega^{*}}{\Gamma_{31}\left[\Gamma_{31}\Gamma_{12}\Gamma_{13}\Gamma_{23}+\left(\Gamma_{31}\Gamma_{13}+\Gamma_{13}\Gamma_{23}\right){\left|\Omega \right|}^{2}\right]},$$

(A6e) $$B_{S}=-i\frac{N_{13}\Gamma_{32}\Omega_{\mathrm{PF}}\Omega +N_{23}\Gamma_{23}\Omega_{\mathrm{PF}}\Omega }{\Gamma_{31}\Gamma_{12}\Gamma_{32}\Gamma_{23}+\left(\Gamma_{31}\Gamma_{32}+\Gamma_{32}\Gamma_{23}\right){\left|\Omega \right|}^{2}},$$

(A6f) $$B_{S^{\prime }}=-i\frac{N_{13}\Gamma_{13}\Omega_{\mathrm{PB}}\Omega +N_{23}\Gamma_{31}\Omega_{\mathrm{PB}}\Omega }{\Gamma_{31}\Gamma_{21}\Gamma_{13}\Gamma_{32}+\left(\Gamma_{13}\Gamma_{31}+\Gamma_{31}\Gamma_{32}\right){\left|\Omega \right|}^{2}},$$

(A6g) $$B_{C}=-i\frac{N_{13}\Gamma_{13}\Omega_{\mathrm{PF}}\Omega +N_{23}\Gamma_{31}\Omega_{\mathrm{PF}}\Omega }{\Gamma_{31}\Gamma_{21}\Gamma_{13}\Gamma_{32}+\left(\Gamma_{13}\Gamma_{31}+\Gamma_{31}\Gamma_{32}\right){\left|\Omega \right|}^{2}},$$

(A6h) $$B_{C^{\prime }}=-i\frac{N_{13}\Gamma_{32}\Omega_{\mathrm{PB}}\Omega +N_{23}\Gamma_{23}\Omega_{\mathrm{PB}}\Omega }{\Gamma_{31}\Gamma_{12}\Gamma_{32}\Gamma_{23}+\left(\Gamma_{31}\Gamma_{32}+\Gamma_{32}\Gamma_{23}\right){\left|\Omega \right|}^{2}}.$$
